# Latent Tuberculosis Screening Using Electronic Health Record Data

**DOI:** 10.3201/eid2609.191391

**Published:** 2020-09

**Authors:** Jeffrey D. Jenks, Richard S. Garfein, Wenhong Zhu, Michael Hogarth

**Affiliations:** University of California, La Jolla, California, USA

**Keywords:** latent tuberculosis infection, epidemiology, tuberculosis, screening, tuberculosis and other mycobacteria, respiratory infections, bacteria, California, rifampin, electronic health records

## Abstract

Screening for latent tuberculosis infection is recommended for foreign-born persons in the United States. We used proxy data from electronic health records to determine that 17.5% of foreign-born outpatients attending the UC San Diego Health clinic (San Diego, CA, USA) underwent screening. Ending the global tuberculosis epidemic requires improved screening.

The World Health Organization End TB Strategy aims to end the global tuberculosis (TB) epidemic by 2035 (*1*). The US Preventive Service Task Force (*2*) and Centers for Disease Control and Prevention (*3*) recommend screening for latent tuberculosis infection (LTBI) in populations at increased risk for infection or progression to TB disease, including foreign-born persons and former residents of countries with increased TB prevalence. Seventy-four percent of active TB cases in San Diego County, California, USA, occur among foreign-born persons, most of whom are from the Philippines, Vietnam, and Mexico; 80% result from reactivated LTBI (*4*). Therefore, TB elimination in the United States requires better diagnosis and treatment of LTBI, especially in foreign-born persons in areas with a low background prevalence of TB, such as San Diego County. However, the frequency of screening for LTBI in foreign-born persons is unknown. 

Because medical records often lack information about country of birth, we assessed whether self-reported nationality plus preferred language is a good proxy variable for foreign birth. We used this proxy to determine LTBI screening, prevalence, and treatment rates in foreign-born persons seen at UC San Diego Health (UCSDH) Medical Center in San Diego. We searched the electronic health record (EHR) at UCSDH and validated this search by reviewing a subset of individual EHRs. The University of California San Diego Institutional Review Board approved this study.

We used the clinical data repository module of our EHR, EPIC (https://www.epic.com), to search the records of all patients who accessed care in the outpatient clinic at UCSDH at least once from March 31, 2018, through March 30, 2019, and who were determined to be at high risk for LTBI on the basis of birth country (*5*). We calculated the proportion of foreign-born persons screened for LTBI from the total number who met our search criteria, and we compared results using a χ^2^ test with a 2-sided p value of <0.05. Self-reported nationality and preferred language was used as a proxy for birth country. For example, we used Mexican nationality and Spanish language as a proxy for being born in Mexico. 

A total of 8,234 persons met our search criteria, most of whom were female, were Mexican, and identified Spanish as their primary language (Table). Overall, 1,437 (17.5%) underwent LTBI screening while receiving care at UCSDH, most with the Quantiferon-TB Gold test (QIAGEN, https://www.qiagen.com). Detailed review of 250 randomly selected patient EHRs from persons who underwent LTBI screening found that 209 (83.6%) had documentation of being born, living, or spending a considerable amount of time (including frequent travel) in a TB-endemic country. A higher proportion of men (19.3%) than women (16.4%) had been screened for LTBI; otherwise, persons who were and were not screened did not differ significantly. Of those screened for LTBI, 956 (66.5%) tested negative and 379 (26.4%) positive by tuberculin skin test or Quantiferon-TB Gold test. To validate LTBI status, we reviewed 250 randomly selected EHRs of patients screened for LTBI, of whom 174 (69.6%) were determined not to have LTBI, 73 (29.2%) had newly diagnosed LTBI, and 3 (1.2%) had pulmonary TB.

**Table:** TB testing and treatment among foreign-born outpatients attending UC San Diego Health Medical Center, San Diego, California, USA, March 31, 2018–March 30, 2019*

Variable	TB tested, no. (%)		p value
	No, n = 6,797	Yes, n = 1,437	
Sex			
F	4,390 (64.6)	860 (59.8)	0.000
M	2,407 (35.4)	577 (40.2)	
Nationality or regionality			
Mexican	5,142 (75.7)	1,074 (74.7)	1.0
Chinese	732 (10.8)	186 (12.9)	
Vietnamese	635 (9.3)	91 (6.3)	
Filipino	186 (2.7)	44 (3.1)	
Guatemalan	43 (0.6)	12 (0.8)	
Asian Indian	18 (0.3)	6 (0.4)	
African	13 (0.2)	10 (0.7)	
Other	17 (0.3)	10 (0.7)	
Unknown	11 (0.2)	4 (0.3)	
Preferred language			
Spanish	5,215 (76.7)	1,094 (76.1)	0.097
Vietnamese	646 (9.5)	90 (6.3)	
Mandarin	384 (5.6)	108 (7.5)	
Chinese	265 (3.9)	68 (4.7)	
Tagalog	184 (2.7)	44 (3.1)	
Cantonese	82 (1.2)	15 (1.0)	
African†	11 (0.2)	12 (0.8)	
Telugu	2 (<1.0)	1 (<1.0)	
Haitian Creole	0	5 (0.3)	
Country of birth			
Unknown	6,745 (99.2)	1,362 (94.8)	1.0
Mexico	49 (0.7)	66 (4.6)	
Other	3 (<1.0)	9 (0.6)	
TB test			
QuantiFERON-TB Gold test‡	NA	1,340 (93.2)	NA
TST	NA	97 (6.8)	
TB test result			
Negative	NA	956 (66.5)	NA
Positive§	NA	379 (26.4)	
Equivocal/low mitogen	NA	102 (7.1)	

*Foreign-born is a proxy for outpatients at UC San Diego Health Medical Center with a self-reported nationality of Mexican, Chinese, Vietnamese, Filipino, Guatemalan, Asian Indian, African plus preferred language other than English. NA, not applicable, TB, tuberculosis; TST, tuberculin skin test.  †Includes Amharic, Somali, and Swahili. ‡QIAGEN, https://www.qiagen.com.  §QuantiFERON TB-Gold titer >0.35 IU/mL or reactive TST considered positive.

To determine the proportion of patients who had LTBI, we searched the EHRs of the 8,234 patients for isoniazid, rifampin, and rifapentine prescription patterns. This search identified 184 patients who had been prescribed rifampin or isoniazid and either had completed or were still undergoing treatment. To validate the EHR search we reviewed these records. A total of 135 (73.4%) patients had been treated for LTBI and 28 (15.2%) had been or were being treated for active pulmonary TB or an atypical mycobacterial infection. The remaining 23 (11.4%) had been prescribed isoniazid or rifampin for another reason, had previously been treated for LTBI and isoniazid or rifampin was documented as a historical medication, or refused treatment. No patients had been prescribed rifapentine. Of those who began treatment for LTBI, 101 (74.8%) completed or were still undergoing treatment at the time of the study, 5 (3.7%) stopped treatment, and treatment completion was unknown for 29 (21.6%).

In our tertiary/quaternary medical center, which serves a large population of foreign-born patients, we found self-reported nationality and preferred language to be a good proxy for foreign-born persons and others who meet the US Preventive Service Task Force and Centers for Disease Control and Prevention guidelines for LTBI screening. However, our single-center study is in a unique setting and so might not reflect findings in other settings. Our proposed screening strategy might miss persons who prefer speaking English but would otherwise meet criteria for LTBI screening. This study identified missed opportunities for screening and diagnosis of LTBI among foreign-born persons; of those who had a recent diagnosis of LTBI, most were successfully treated. Improved LTBI screening, possibly with the use of routine EHR tools, is needed to end the global TB epidemic.
